# Can Procalcitonin Be Dosed in Bovine Milk Using a Commercial ELISA Kit?

**DOI:** 10.3390/ani12030289

**Published:** 2022-01-25

**Authors:** Valentina Meucci, Chiara Orsetti, Micaela Sgorbini, Federica Battaglia, Marta Cresci, Francesca Bonelli

**Affiliations:** 1 Department of Veterinary Science, University of Pisa, Via Livornese snc, 56122 Pisa, Italy; valentina.meucci@unipi.it (V.M.); chiara.orsetti@phd.unipi.it (C.O.); micaela.sgorbini@unipi.it (M.S.); federica.battaglia@phd.unipi.it (F.B.); martacresci@gmail.com (M.C.); 2 Centro di Ricerche Agro-Ambientali “E. Avanzi”, University of Pisa, Via Vecchia di Marina 6, 56122 Pisa, Italy

**Keywords:** procalcitonin, bovine, mastitis, milk, plasma, ELISA kit

## Abstract

**Simple Summary:**

Mastitis is one of the major economic and animal welfare problems on dairy farms. The gold standard test for mastitis diagnosis is milk culture, but bacteria are not always isolated (only in 11–44% of milk samples from clinical cases of mastitis) and sometimes a positive culture can result from a contamination of the milk. Procalcitonin is a new biomarker which may lead to an early detection of inflammation due to bacterial infection. In humans, procalcitonin concentration has also been evaluated in milk in addition to plasma. The authors aimed to evaluate the possible application of a commercially available ELISA kit for bovine procalcitonin for the assessing of procalcitonin in bovine milk samples. Plasma and milk samples from cows with mastitis were collected to measure procalcitonin concentrations by using a bovine procalcitonin ELISA kit. Our results showed that the ELISA kit tested can be employed to assess bovine procalcitonin in plasma but not for analyzing milk samples.

**Abstract:**

The aim was to evaluate the use of a bovine procalcitonin (PCT) ELISA kit (Cusabio, China) for assessing PCT in bovine milk samples. Validation was performed by using 10 plasma and corresponding milk samples from mastitic cows. The limit of detection (LOD) was calculated. The coefficient of variation (CV%) of the readings of five plasma samples measured five times in the same plate (intra-assay) and the CV% of the same five samples read five times in three separate plates was evaluated. Parallelism was determined by serial twofold dilutions of five plasma and corresponding milk samples. Milk samples were analyzed with and without centrifugation. Regarding plasma PCT, the method presented an inter- and intra-CV < 23.7% and parallelism had very good recovery values. The ELISA kit studied can measure bovine plasma PCT concentrations. The kit antibodies fail in binding PCT in milk samples because all centrifuged milk samples showed a lower LOD than blank samples. Only three uncentrifuged milk samples showed measurable PCT concentrations. Due to these results, the commercial ELISA kit investigated could not be employed for the detection of PCT in milk samples.

## 1. Introduction

Mastitis is still considered the principal disease affecting dairy cows, despite the massive work done by national control programs to implement surveillance and therapy of this condition [1]. Diagnosis of mastitis can be achieved by different techniques, but milk culture represents the “gold standard.” Unfortunately, in 11 to 44% of milk samples from cows with clinical mastitis, bacteria were not isolated [2,3] or samples tested positive due to the colonization of the teat canal and cistern with no major involvement of the mammary parenchyma [4]. Recently, there has been an increased interest in finding new markers, such as haptoglobin, serum amyloid A [5,6], and blood serum proteins [7], which may lead to an early detection of inflammation due to bacterial infection [8,9,10,11].

Procalcitonin (PCT), the prohormone of calcitonin, is a glycoprotein consisting of 116 amino acids with 13 kDa molecular weight [12]. Procalcitonin production is driven by the calcitonin gene-1 (CALC-1). Normally, the transcription of the CALC-1 gene is principally restricted to thyroidal activity for calcitonin synthesis; thus, there is almost no PCT circulate in the body system [13]. During bacterial infections, plasmatic PCT levels increase because lipopolysaccharides (LPS) and the inflammatory cytokines specific to bacterial infection, such as interleukin-1 beta (IL-1β), IL-6 and tumor necrosis factor-alpha (TNF-α), stimulate the upregulation of the expression of the CALC-1 gene in multiple tissues, leading to a massive production of PCT from many tissues [12,14]. In veterinary medicine, PCT has been investigated as a biomarker of bacterial infections in adult horses [15,16,17,18,19,20,21,22], foals [23,24,25], and dogs [26,27,28,29], while few studies have been conducted in ruminants [30,31,32,33]. Two papers evaluated plasma PCT concentrations in septic neonatal calves [30,31], and in feedlot calves (4–12 months old) with respiratory disease [32]. In both cases PCT plasma concentrations were significantly higher in sick calves compared with healthy ones. A very recent study found that serum PCT was higher in cows affected by subclinical mastitis [33].

Milk will represent a better biological sample for the diagnosis of mastitis in cows, because it is easier to take in field condition and sampling requires a less invasive procedure for cows. Struck et al. [34] were able to quantify PCT in the milk of breastfeeding mothers, showing that the kinetic profile of PCT in milk was similar to other proinflammatory cytokines.

Currently, PCT in animals can be dosed by using enzyme-linked immunosorbent assays (ELISA); however, available kits for bovine PCT are not validated for milk samples. Our aim was to evaluate the possible use of a commercially available ELISA kit marketed for bovine procalcitonin for the evaluation of PCT in bovine milk samples.

## 2. Materials and Methods

### 2.1. General and Ethical Animal Care

The present study has been approved by the dedicated Committee of the University of Pisa (2825/2014). Sample collection took place in 2018 at the university farm (Centro di Ricerche Agro-Ambientali “E. Avanzi”), while laboratory analysis was performed at the Laboratory of Veterinary Pharmacology and Toxicology of the Department of Veterinary Sciences, University of Pisa, Italy.

### 2.2. Animals

Plasma and milk samples from 10 sick cows were collected to measure PCT concentrations. All animals were raised in the same environment and under the same feeding regimen. Briefly, cows calved all year round. Two different kinds of bull semen were used: heifers and first lactation cows received sexed semen from an Italian Friesian bull, while multiparous cows received semen from Limousine bull. This is made because of the higher market price of mixed calves. Heifers born from sexed semen were raised at the farm as a rearing herd. Cows were housed in a free-stall barn with straw used as bedding material, changed twice per week with daily adding of clean straw. The total mixed ration was given to all the cows in lactation twice a day; moreover, animals had ad libitum access to fresh water. The farm was equipped with a Herringbone milking parlor; animals were milked twice a day at 5 a.m. and 4 p.m.

The average parity and lactation weeks were 2.6 ± 1.1 and 36.4 ± 12.9, respectively. Cows were considered affected by clinical mastitis and included in the present study based on the following criteria: (1) signs of inflammation (redness, swelling, and pain) at quarter level; (2) the California mastitis test was ≥1 [35] and the somatic cell count was ≥250,000 cells/mL [36]. Cows affected by diseases other than clinical mastitis (e.g., lameness or rumen problems) and with a history of clinical or subclinical mastitis the month before sampling were excluded from the protocol.

At the time of the diagnosis, blood samples from mastitic cows were taken using a sterile syringe and a heparinized tube (FL Medical, Torreglia, Italy). Samples were immediately centrifuged at 3000 rpm × 10 min (Legend RT, Sorvall; ThermoFisher Scientific Inc., Waltham, MA, USA). Plasma samples were then divided in 5 aliquots, which were stored at −80 °C from the time of collection and thawed just before use. Contextually, a 15 mL milk sample from the affected quarter was taken in a sterile way and put inside a dedicated tube (Falcon^®^, BD, Italy). Milk was stored at −80 °C until the time of analysis. All the samples were processed within 3 months.

### 2.3. Procalcitonin ELISA Kit

The kit utilizes a sandwich enzyme immunoassay technique. The reagents’ preparation, incubations, and washes followed the manufacturer’s guidelines.

The kit provided a standard solution which was diluted to yield 1250, 625, 312.5, 156.25, 78.12, and 39.6 pg/mL solutions, with a final blank well containing only the standard diluent (0 pg/mL PCT).

The optical density (OD) of the samples was assessed with a microplate reader set to 450 nm. The OD of the blank well was subtracted from each sample OD. The standard curve was built by plotting the mean absorbance of standards against the known concentration of standards, leading to the calculation of the PCT concentration in samples. The calibration curve and the characteristics of the kit used (LOD; Calibration Range; Intra-assay and Inter-assay variation) were provided by the manufacturer for plasma, serum, and tissue homogenates (Table 1).

### 2.4. Procalcitonin ELISA Kit Validation

Plasma and corresponding milk samples (*n* = 10) obtained from mastitic cows were used to validate the ELISA kit.

Limit of detection (LOD) of the assay was assessed using the following formula:

(Ablank + 3SDblank), where A is the absorbance of the blank sample and SD is the standard deviations of the absorbance of the blank. Dilution buffer is pipetted into blank wells.

Intra- and inter-assay precision were assessed as the coefficient of variation (CV%) of the readings of 5 plasma samples (cows number 1–5) measured 5 times in the same plate (within run) and the CV% of the same 5 samples read 5 times in three separate plates (run to run). The acceptance criteria were set at <25%.

Serial twofold dilutions of 5 plasma (cows number 6–10) and corresponding milk samples were used to evaluate parallelism and the recovery of the assay. Acceptance criteria were a recovery within 80% and 120%.

Milk samples were analyzed both with and without centrifugation. Milk samples were centrifuged for 15 min at 15,000 *g* and at 4 °C. The supernatant was removed, and the defatted milk was recovered and used for the analysis.

### 2.5. Statistical Analysis

Graph Pad Prism v. 8 software (GraphPad Software, San Diego, CA, USA) was used to perform statistical analysis. The Kolmogorov–Smirnov test was used to evaluate data distribution. Correlation between PCT concentrations in plasma and milk samples was assessed by using the Spearman test. Results in which *p* < 0.05 were considered statistically significant.

## 3. Results

### 3.1. Plasma Samples

The plasma PCT concentrations in samples of analyzed cows are reported in Table 2.

No correlation was found between plasma and milk samples. The LOD has been calculated as 40 pg/mL.

The method showed an intra- and inter-assay coefficient of variation (CV) for PCT in the five plasma samples ranging from 0.05 to 10.4% and from 4.2 to 23.7%, respectively. Parallelism for the five plasma samples showed recovery values ranging from 95.8 to 116.6%. For detailed information please see Supplementary Material (Tables S1–S3).

### 3.2. Milk Samples

The method showed an inter- and intra-coefficient of variation (CV) for PCT in three uncentrifuged milk samples of 65.3%, 52.4%, 60.0% and 45.2%, 58.0%, and 65.2%, respectively; the other two samples had PCT levels < LOD. Parallelism for the milk samples could not be calculated because the samples showed concentrations <LOD. All milk samples analyzed after centrifugation showed PCT concentrations <LOD (Table 2).

## 4. Discussion

In human medicine, PCT has been used as a marker for sepsis diagnosis, but also for controlling the course of the disease, and for antibiotic stewardship [14]. The same use has also been investigated in veterinary medicine [15,16,17,18,19,20,21,22,23,24,25,26,27,28,29,30,31,32,33]. Although there are promising results from previous mentioned studies, in veterinary medicine PCT can be assessed only using ELISA kits from different manufacturers conceived for research purposes. Unfortunately, none of these assays are designed for evaluating PCT concentration in bovine milk.

To our knowledge, there are only a few studies on ruminants’ PCT and none of them have evaluated PCT concentrations in milk. In a study from 2016, a human ELISA kit was employed to detect serum PCT concentrations in neonatal calves affected by colibacillosis, but no validation data were reported [30]. Bonelli et al. [26] measured the plasma PCT concentration in calves using another bovine ELISA kit (Mybiosource, San Diego, CA, USA), showing a partial validation, while El-deeb et al. [32,33] used the same ELISA kit for measuring plasma PCT concentration in calves without showing data on validation. A recent study evaluated PCT levels in serum samples of cows affected by Staphylococcal mastitis [33]. Unfortunately, no information was given by the authors regarding their validation analysis. Thus, although the work could be considered interesting for our research topic, a direct comparison is not possible.

The assay investigated in this study is marketed for the detection of bovine PCT in plasma, serum, and tissue homogenates. However, measuring markers such as PCT in milk, rather than in plasma or serum, would be more feasible under field conditions. In the paper of Struck and colleagues [34], PCT was dosed in the milk of breastfeeding mothers by using a ultra-sensitive two-site immunoluminometric assay in coated tube format (LUMItestI ProCa-S, B.R.A.H.M.S). Thus, our hypothesis involved the possibility of assessing PCT concentrations in milk.

Regarding plasma PCT evaluation, the method presented an inter- and intra-coefficient of variation (CV) for PCT in the five plasma samples of <23.7%. Parallelism, determined by serial twofold dilutions of plasma samples with a high endogenous PCT level had very good recovery values. Thus, the bovine procalcitonin ELISA kit^®^ (Cusabio, Wuhan, China) can measure bovine plasma PCT concentrations.

Struck et al. [34] showed that PCT concentrations in human milk samples were 100 times higher than in the corresponding serum samples. The present results showed acceptable validation data for bovine plasma but not for milk both in centrifuged and not centrifuged samples. These results did not recommend the use of the studied ELISA kit for the measurement of PCT concentrations in milk samples. Procalcitonin could be dosed in humans’ milk probably because the method used was different [34], or this could be due to physiological differences between human and bovine species.

A limitation of this study was the lack of a gold standard test for measuring bovine PCT. Hence, the ELISA kit under investigation could not be compared to a reference method. Furthermore, recombinant bovine PCT protein purified for use in generating and validating a bovine-specific quantitative PCT ELISA is not present, and its sequence is lacking at this moment. Our research demonstrates that PCT concentrations could not be evaluated in bovine milk by using this specific assay; however, the development of different laboratory methods may result in an appropriate processing of the milk matrix for assessing PCT.

## 5. Conclusions

This study supports the hypothesis that the Cusabio bovine PCT ELISA kit is suitable for measuring PCT in bovine plasma samples, with an intra-assay and inter-assay CV of less than 20% and adequate recovery, but the same kit could not be considered a valid system for analyzing milk samples.

## Figures and Tables

**Table 1:** Performance characteristics of studied kit. Legend: CV—coefficient of variation; LOD—limit of detection.

Kit	Type of Sample Suitable for Analysis	Calibration Range	Intra- and Inter-Assay CV	LOD
Bovine PCT	Serum, plasma, tissue homogenates.	39.0–2500.0 pg/mL	<8.0%–<10.0%	9.77 pg/mL

**Table 2:** PCT concentrations (pg/mL) in sick cows measured with bovine PCT ELISA kit. Legend: LOD—limit of detection.

Sample	Plasma	Milk	Milk after Centrifugation
1	410.3	<LOD	<LOD
2	196.1	112.3	<LOD
3	565.4	<LOD	<LOD
4	487.4	<LOD	<LOD
5	785.7	88.6	<LOD
6	381.0	58.7	<LOD
7	282.9	<LOD	<LOD
8	886.5	<LOD	<LOD
9	472.8	<LOD	<LOD
10	160.6	<LOD	<LOD

## Data Availability

The data will be made available if the corresponding author is emailed.

## Supplementary Materials

The following supporting information can be downloaded at: https://www.mdpi.com/article/10.3390/ani12030289/s1, Table S1: Intra-assay (Within-Run) precision of bovine PCT kit; Table S2: Inter-assay (Run to run) precision of bovine PCT kit; Table S3: Linearity of plasma samples serially diluted with dilution Buffer and assayed.
