# Supplementary material for: Can Procalcitonin Be Dosed in Bovine Milk Using a Commercial ELISA Kit?
Source: Animals (Basel). 2022 Jan 25;12(3):289. doi: 10.3390/ani12030289 (PMC8833620; doi:10.3390/ani12030289)
Supplement: Supplementary file 1 [file animals-12-00289-s001.zip › animals-1559933-supplementary.pdf]

Supplementary Material Manuscript number animals-1559933

Table S1. Intra-assay (Within-Run) precision of bovine PCT kit. Legend: SD – standard deviation; CV – coefficient of variation.

Sample	Number of Values	Mean	SD	CV
1	5	410.3	35.4	8.6
2	5	196.1	4.1	2.1
3	5	565.4	58.7	10.4
4	5	487.4	0.26	0.05
5	5	785.7	38.8	4.9

Table S2. Inter-assay (Run to run) precision of bovine PCT kit. Legend: SD – standard deviation; CV – coefficient of variation.

Sample	Number of Values	Mean	SD	CV
1	25	461.9	76.1	16.5
2	25	227.0	36.0	15.9
3	25	624.6	147.6	23.7
4	25	537.5	59.8	11.1
5	25	806.7	33.7	4.2

Table S3. Linearity of plasma samples serially diluted with dilution Buffer and assayed.

Sample	Dilution	Observed (pg/mL)	Expected (pg/mL)	Recovery O/E (%)
6	-	381.0		
	2X	191.3	190.5	100.4
	4X	94.3	95.6	98.9
7	-	282.9		
	2X	137.1	141.5	96.8
	4X	81.8	68.5	119.4
8	-	886.5		
	2X	517.8	443.2	116.6
	4X	262.5	273.9	95.8
9	-	472.8		
	2X	247.7	236.1	104.9
	4X	122.4	123.8	98.8
10	-	160.6		
	2X	86.1	80.3	107.1
	4X	44.2	43.0	102.3
